# Transcriptomics Reveal Altered Metabolic and Signaling Pathways in Podocytes Exposed to C16 Ceramide-Enriched Lipoproteins

**DOI:** 10.3390/genes11020178

**Published:** 2020-02-07

**Authors:** Samar M. Hammad, Waleed O. Twal, Ehtesham Arif, Andrea J. Semler, Richard L. Klein, Deepak Nihalani

**Affiliations:** 1 Department of Regenerative Medicine and Cell Biology, Medical University of South Carolina, Charleston, SC 29425, USA; twalwo@musc.edu; 2 Division of Nephrology, Department of Medicine, Medical University of South Carolina, Charleston, SC 29425, USA; arif@musc.edu (E.A.); nihalani@musc.edu (D.N.); 3 Division of Endocrinology, Metabolism, and Medical Genetics, Department of Medicine, Medical University of South Carolina, Charleston, SC 29425, USA; semleraj@musc.edu (A.J.S.); kleinrl@musc.edu (R.L.K.); 4 Research Service, Ralph H. Johnson Department of Veterans Affairs Medical Center, Charleston, SC 29401, USA

**Keywords:** RNA-Seq, podocyte, ceramide, lipoprotein, sphingolipids, mTOR

## Abstract

Sphingolipids are bioactive lipids associated with cellular membranes and plasma lipoproteins, and their synthesis and degradation are tightly regulated. We have previously determined that low plasma concentrations of certain ceramide species predict the development of nephropathy in diabetes patients with normal albumin excretion rates at baseline. Herein, we tested the hypothesis that altering the sphingolipid content of circulating lipoproteins can alter the metabolic and signaling pathways in podocytes, whose dysfunction leads to an impairment of glomerular filtration. Cultured human podocytes were treated with lipoproteins from healthy subjects enriched in vitro with C16 ceramide, or D-erythro 2-hydroxy C16 ceramide, a ceramide naturally found in skin. The RNA-Seq data demonstrated differential expression of genes regulating sphingolipid metabolism, sphingolipid signaling, and mTOR signaling pathways. A multiplex analysis of mTOR signaling pathway intermediates showed that the majority (eight) of the pathway phosphorylated proteins measured (eleven) were significantly downregulated in response to C16 ceramide-enriched HDL2 compared to HDL2 alone and hydroxy ceramide-enriched HDL2. In contrast, C16 ceramide-enriched HDL3 upregulated the phosphorylation of four intermediates in the mTOR pathway. These findings highlight a possible role for lipoprotein-associated sphingolipids in regulating metabolic and signaling pathways in podocytes and could lead to novel therapeutic targets in glomerular kidney diseases.

## 1. Introduction

Sphingolipids are both structural lipids and signaling molecules with significant physiological functions. Sphingolipids are found associated with cellular membranes and plasma lipoproteins, and their synthesis and degradation are tightly regulated to maintain homeostasis [1,2]. A classic form of chronic renal disease, Fabry disease, results from the genetic impairment of the glycosphingolipid metabolism [3]. Furthermore, diseases associated with kidney dysfunction—including diabetic nephropathy, polycystic kidney disease, and renal cell carcinoma—have been associated with changes in renal glycosphingolipid levels and the associated metabolism [4,5,6]. However, little is known about how changes in plasma sphingolipids and the sphingolipid composition of lipoproteins may contribute to the development of renal disease. Urine protein metabolites in patients with diabetic kidney disease provided some biochemical insights for identifying novel candidate biomarkers for the disease [7]; however, the information regarding plasma and urine lipidomics is still scarce. Sphingolipids are rapidly emerging as clinically important biomolecules due to their effects on multiple metabolic pathways [1]. Unlike other lipid molecules (e.g., cholesterol) small changes in the levels of sphingolipids can elicit potent responses and it is becoming increasingly clear that dysfunctional lipid metabolism extends far beyond cholesterol and triglycerides [8,9].

There is growing evidence that sphingolipid metabolism is altered in diabetes [10,11]; however, the majority of evidence comes from studies in animal models [12,13,14] with little attention given to the delivery of plasma sphingolipids to renal cells. We previously showed that sphingolipidomics can predict the risk of developing nephropathy in type 1 diabetes patients [15]. We measured plasma concentrations of ceramide species to investigate their association with the development of albuminuria in type 1 patients in the Diabetes Control and Complications Trial/Epidemiology of Diabetes Interventions and Complications (DCCT/EDIC) study during 14–20 years of follow-up [15]. The results demonstrated that increased plasma levels of C16 ceramide and very long (C20, C20:1, C22:1, C24:1, C26, and C26:1) chain ceramide species measured at DCCT baseline were associated with a decreased likelihood of developing macroalbuminuria [15]. Other investigators found higher levels of ceramide in HDL particles of obese patients who were insulin insensitive [13]; in the small HDL particles (HDL3) of type 2 diabetes patients with dyslipidemia [16], and in HDL after ingestion of a high fat diet [17].

**Figure 1:**
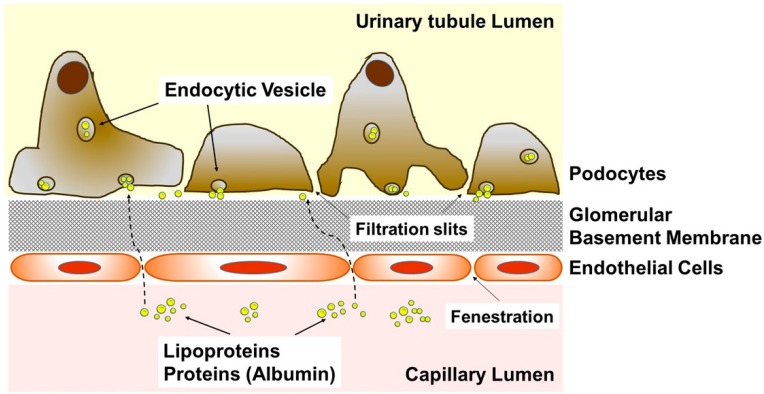
Cartoon depicting the microstructure of the glomerulus. Diagram showing the transfer of albumin and lipoproteins including their sphingolipid cargo from the capillary lumen through fenestration of endothelial cells to the podocytes.

Ceramides are an integral part of cell membranes associated with macrodomains regulating surface receptors and signaling pathways [18,19,20,21,22,23]. Previous studies addressing the role of ceramide in diabetes focused on insulin resistance [12,13,24,25] and did not address diabetic complications including nephropathy. There is an increasing body of evidence suggesting that injury to podocytes leads to proteinuria and lipiduria (lipuria) in nephropathy [26,27,28] (Figure 1). In this study, we exposed cultured human podocytes to C16 ceramide-enriched lipoproteins (low density lipoproteins (LDL), high-density lipoproteins 2 and 3 (HDL2 and HDL3) and performed a comprehensive transcriptomics analysis. The results revealed possible metabolic and signaling pathways through which extracellular sphingolipids carried on lipoprotein particles can regulate the expression of genes encoding podocyte functionality and pathophysiology. Special focus is on the mTOR signaling pathway, which is well known to regulate the expression of slit diaphragm proteins and cytoskeleton structure in podocytes [29,30,31].

## 2. Materials and Methods

### 2.1. Cells and Reagents

Human immortalized podocytes were originally obtained from Dr. Moin Saleem [32]. Tissue culture flasks and plates were obtained from Greiner bio-one, Inc., and tissue culture reagents were from Gibco. Lipoproteins were isolated from healthy volunteers as per our routine procedure [33]. Prior to enrollment in the study, written informed consent was obtained from each volunteer. Blood collection for lipoprotein preparation was approved by the ethics committee of the Medical University of South Carolina (MUSC) and was performed according to the protocols approved by our institutions’ institutional review board (IRB), (Protocol number: Pro00070819). All volunteer data were analyzed anonymously. Blood was collected after a 12 h fast from healthy subjects who were normolipemic, not receiving prescription medication for any acute or chronic condition, and without family history of coronary artery disease, peripheral vascular disease, or stroke; none of the subjects were receiving antioxidant therapy. The blood was pooled from 3 or 4 donors in the presence of a lipoprotein preservative cocktail consisting of ethylenediaminetetraacetic acid (EDTA) (0.1% *w/v*), chloramphenicol (20 µg/mL), gentamycin sulfate (50 µg/mL), epsilon amino-caproic acid (0.13% *w/v*), and dithiobisnitrobenzoic acid (0.04% *w/v*) to inhibit the activity of lecithin-cholesterol acyltransferase, a major enzyme on HDL and LDL that converts free cholesterol into cholesteryl ester. Blood was then centrifuged, plasma was obtained, and lipoproteins fractioned by preparative ultracentrifugation as described previously [33]. Lipoprotein fractions were dialyzed against saline/EDTA (150 mM NaCl, 300 µM EDTA, pH 7.4), sterilized by filtering through a 0.22 µm membrane, and stored at 4 °C until used. Protein levels in lipoprotein preparations were measured using the Lowry method.

Podocytes were plated at 0.5–0.6 × 10^6^ cell in 10-cm^2^ collagen-treated plates in RPMI 1640 media supplemented with 10% fetal bovine serum (FBS) (Corning), 200 units/mL Penicillin/Streptomycin (Roche Applied Science), and 1% insulin-transferrin-sodium selenite (ITS) (Sigma), and incubated overnight at 33 °C, 5% CO2. Media was then changed but without the inclusion ITS, and cells were transferred to a 37 °C-incubator. Cells were grown for 14 days to be differentiated and the growth media were changed every 3–4 days.

### 2.2. Ceramide Enrichment of Lipoprotein Particles

Stock solution of each lipoprotein: LDL, HDL2 and HDL3 [2 mL of 4113 µg protein/mL in phosphate buffered saline (PBS)] was prepared [33] and used fresh for enrichment procedure. We have selected C16 and C24 ceramide species as representatives of long and very long chain ceramide species, respectively, and because they were previously shown to be negatively associated with the likelihood to develop macroalbuminuria in type 1 diabetes patients [15]. Ceramides were synthesized at the Synthesis Unit of the MUSC Lipidomics Shared Resource. Stock solutions of sphingolipids: D-e-C16 ceramide (C16 ceramide, MW 538.2), D-e-C24 ceramide (C24 ceramide, MW 650.2), and D-e- hydroxy C16 ceramide (2OH C16-Ceramide, MW 554) were prepared in 100% methanol at 100 µM each.

Duplicate aliquots from each lipoprotein (100 µg, 24 μL each) were analyzed at the Analytical Unit of the MUSC Lipidomics Shared Resource for sphingolipid content as previously described [33]. All preparations were performed in foil wrapped sterile glass tubes under yellow light. To each sterile tube, 50 µL of each synthetic ceramide solution was added and evaporated onto the side of the tube using nitrogen. The remaining volume of each 2 mL sterile lipoprotein solution with additional 50 µL of each ceramide solution were added to the tube. Each tube was then sealed under nitrogen and incubated for 24 h at 37 °C. Duplicate aliquots of each ceramide-enriched lipoprotein (100 µg, 24 µL each) were frozen in glass tube until analysis at the MUSC Lipidomics Shared Resource. The enriched lipoprotein solution was dialyzed against 0.01% EDTA in PBS, and aliquots (100 µg) of each ceramide-enriched lipoprotein were analyzed for sphingolipid content. Control (native) lipoproteins were not supplemented with ceramide but were subjected to a similar processing protocol.

### 2.3. RNA Sequencing (RNA-Seq) Analysis

Podocytes were differentiated as described above. Media were changed 24 h before the start of the experiment, then lipoproteins with and without enrichment were added to a final concentration of 200 μg/mL. After 7 h of incubation at 37 °C, 5% CO2, the plates were washed with PBS, and stored at −80 °C wrapped with aluminum foil.

RNA was extracted from frozen cells in the stored plates using Qiagen’s RNeasy kit according to manufacturer protocol and concentration quantified. An amount of 100–200 ng of total RNA was used to prepare RNA-Seq libraries using the TruSeq RNA Sample Prep Kit V2 (Illumina, San Diego, CA, USA), following the protocol described by the manufacturer. High-throughput single read (1 × 50 cycles) sequencing (HTS) was performed using an Illumina HiSeq2500 (v4 chemistry) with each sample sequenced to a minimum depth of ~50 million reads. Data were subjected to Illumina quality control (QC) procedures (>80% of the data yielded a Phred score of 30). Secondary analysis was carried out on an OnRamp Bioinformatics Genomics Research Platform (OnRamp Bioinformatics, San Diego, CA, USA) (Codexis, Redwood City, CA, USA) [34,35]. OnRamp’s advanced Genomics Analysis Engine utilized an automated RNAseq workflow to process the data, including (i) data validation and quality control, (ii) read alignment to the human genome (hg19) using TopHat2 [36], which revealed 96.9% mapping of the single end-reads, and (iii) generation of gene-level count data with HTSeq. The resulting SAM files were sorted and inputted into the Python package HTSeq to generate count data for gene-level differential expression analyses [Genomics Research Platform with RNAseq workflow v1.0.1, including FastQValidator v0.1.1a, Fastqc v0.11.3, Bowtie2 v2.1.0, TopHat2 v2.0.9, HTSeq v0.6.0].

In order to infer differential signal within the data sets with robust statistical power, we utilized the paired-T test on Limma [37]. The data was first converted to log2 with the “voom” function and then fitted to a linear model by “lm.fit” [37]. Fold-change (FC) estimation and hypothesis testing for differential expression were performed using the lm.fit and ebays function [37]. The transcript expression data from Limma analysis of the samples were sorted according to their adjusted *p*-value or *q*-value, which is the smallest false discovery rate (FDR) at which a transcript is called significant. FDR is the expected fraction of false positive tests among significant tests and was calculated using the Benjamini–Hochberg multiple testing adjustment procedure. The statistical analysis of pathways and gene ontology terms were carried out using this sorted transcript list as described previously [38,39] and using iPathwayGuide (Advaita Bioinformatics, Plymoth, MI, USA) [40].

### 2.4. Multiplex Analysis of MTOR Signaling Pathway Intermediates

Cells were plated and differentiated in 6-well collagen-treated plates at 3.0 × 10^5^ cells per well as described above. Media were changed 24 h before the start of the experiment, then lipoproteins with and without enrichment were added to a final concentration of 200 μg/mL. After 2 h of incubation at 37 °C, 5% CO2, cells were washed with cold PBS and extracted using the extraction buffer of the mTOR signaling Millipex kit from Millipore (Billerica, MA, USA) with added enzyme inhibitors (Roche). Extracts were stored at −80 °C until use. Cell lysates were cleared by centrifugation at 16,000× *g* for 15 min, then by passing in a Costar 0.22 µm spin-x filter unit (Cambridge, MA, USA). A total of 25 µL of cell extract containing 11.5 µg of protein in assay buffer was used for each Millipex assay well. The Millipex mTOR signaling kit contains eleven antibodies against the following phosphorylated intermediates: GSK3B, IGFR1, IRS1, AKT, mTOR, P70S6K, IR, PTEN, GSK3a, TSC2, and RPS6. The kit antibodies were validated by the manufacturer for lack of cross reactivity. The assay was performed according to the manufacturer’s instructions and using the Biorad Bio-Plex 200 Multiplex System (Bio-Rad) at the MUSC Proteogenomics facility. All treatments were performed in duplicate wells and the cell extract from each well was analyzed in duplicates. Results from treatments with ceramide-enriched lipoprotein were compared to those with control lipoproteins using Student *t*-test at *p* ≤ 0.05.

## 3. Results and Discussion

We previously demonstrated that increased plasma levels of baseline C16 ceramide and very long (C20–C26) chain ceramide species were associated with decreased likelihood to develop macroalbuminuria after several years of follow-up [15]. On the other hand, higher levels of circulating long and very long chain ceramides were reported in systemic lupus erythematous patients with confirmed renal involvement [41]. In the present study, we aimed at determining whether lipoproteins enriched with C16 ceramide species could induce critical metabolic and signaling pathways in cultured human podocytes.

### 3.1. Ceramide Enrichment of Lipoprotein Particles

**Figure 2:**
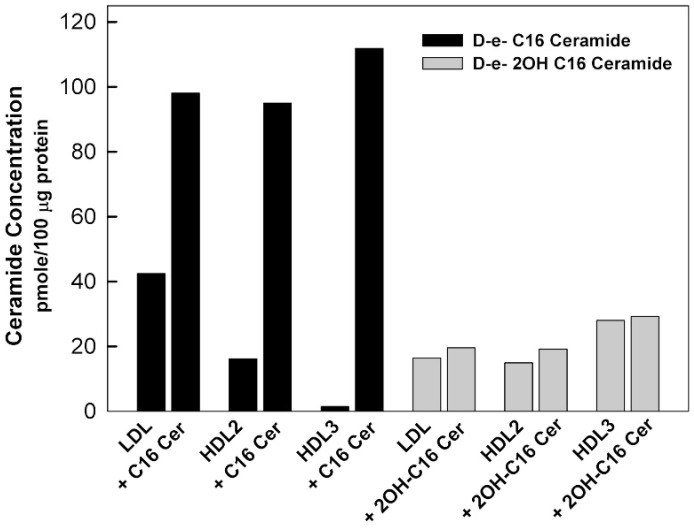
Enrichment of the lipoprotein particles with ceramide. Lipoproteins isolated from healthy volunteers were incubated with 100 μM of ceramides. After incubation for 24 h at 37 °C, the lipoproteins were dialyzed against PBS and samples of before and after dialysis were analyzed for lipoprotein content. D-e-C16 ceramide had the highest incorporation while D-e-2OH C16 ceramide had low incorporation and D-e-C24 ceramide had no incorporation (Data not shown).

We previously determined levels of sphingolipid species in isolated lipoprotein classes in healthy human subjects using mass spectroscopy [33]. The smallest lipoprotein particles, HDL3 were found to be the major carriers of sphingosine 1-phosphate (S1P), dihydrosphingosine 1-phosphate, and sphingosine. HDL3 particles contain the lowest levels of sphingomyelin and ceramide; however, HDL2 and HDL3 particles have similar sphingomyelin/ceramide ratios (72.9% and 78.9%, respectively) despite the difference in their particle size (8.5–13 and 7.3–8.5 nm, respectively) [33]. The results of the analysis of the ceramide species in lipoprotein particles showed that the concentration of C24 ceramide is the highest, followed by C24:1, C 22, C20, C16, and C18 ceramide species [33]. Ståhlman et al. found that small HDL-particles predominated in dyslipidemic subjects, with and without diabetes, compared to respective normolipidemic controls, and were distinguished as the primary carrier of ceramides, which is known for promoting inflammation and insulin resistance [16]. In healthy individuals, LDL particles are typically the major carriers of ceramide compared to VLDL and HDL particles [33,42]. In the current study, when lipoprotein particles were incubated in vitro with different ceramide species, C16 ceramide had the highest level of incorporation into all lipoproteins (LDL, HDL2, HDL3) (Figure 2). 2OH C16 ceramide had lower incorporation (Figure 2), whereas the very long-chain C24 ceramide was not incorporated in any lipoprotein incorporation (Data not shown). In vivo, the main tissue sources for circulating sphingolipids, their flux rate and half-life remain unclear. Clues to the origin of sphingolipids in the circulation have come from the recent studies, which identified microsomal triglyceride transfer protein (MTP) and ATP binding cassette family A protein 1 (ABCA1) as critical determinants of sphingolipid levels in lipoproteins [43,44]. A possible reason why the very long chain ceramide (C24) was not incorporated into the lipoprotein particles in vitro is probably the lack of an active process that requires MTP, similar to the naturally occurring process in the intracellular in vivo system [43].

### 3.2. Signaling and Metabolic Pathways Induced by C16 Ceramide-Enriched Lipoproteins

The signaling and metabolic pathways that were affected by exposing cultured human podocytes to ceramide-enriched lipoproteins for seven hours were examined using RNAseq analysis. The pathways that were significantly (*p* < 0.05) affected by the incubation of ceramide-enriched LDL, HDL2 and HDL3 are presented in Tables S1–S3**,** respectively. Treatment of podocytes with C16 ceramide-enriched LDL showed that the metabolic and signaling pathways were most affected when compared to C16 ceramide-enriched HDL2 or C16 ceramide-enriched HDL3, each relative to its corresponding control (native) lipoprotein. We focused primarily on the pathways that were significantly affected by C16 ceramide-enriched LDL and are pertinant to kidney functions. Thus, the sphingolipid metabolic pathway, glycophospholipid biosynthesis-ganglio series pathway, sphingolipid signaling pathway, adherens junctions pathway, mTOR signaling pathway, focal adhesion pathway, and apopotosis pathway were studied (Table 1).

#### 3.2.1. Sphingolipid Metabolism Pathway

**Figure 3:**
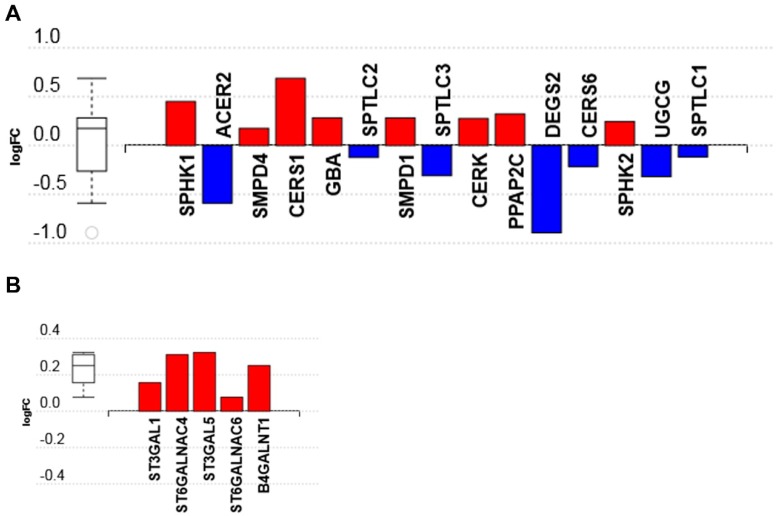
The sphingolipid metabolism mRNA transcripts regulated in response to C16 ceramide-enriched LDL in human podocytes. Podocytes were induced with 200 μg/mL C 16 ceramide-enriched or native LDL and incubated for 7 h at 37 °C, 5% CO2; RNA was extracted and RNA-seq assay was performed as described under Methods. (**A**) Sphingolipid metabolism; (**B**) glycosphingolipids synthesis. Figure generated using iPathwayGuide (Advaita Bioinformatics) software; log FC, logarithm of fold change of gene expression. Box and whisker plot: The ends of the box are the upper and lower quartiles, box spans the interquartile range. Horizontal line inside the box denotes the median and the whiskers are the two lines outside the box extend to the highest and lowest observations.

Our data show that genes regulating the sphingolipid metabolism pathway (Figure S1) partly regulate the sphingolipid signaling pathway (Figure S2). This is not surprising, since several sphingolipid metabolites (e.g., S1P, sphingosine, ceramide, ceramide 1-phosphate) are bioactive signaling molecules. C16 ceramide-enriched LDL induced the upregulation of sphingosine kinase 1 and 2 (*SPHK1 & SPHK2*)*,* ceramide synthase 1 ((*CERS1*)*,* generates C18 ceramide), ceramide kinase (*CERK*). On the other hand, C16 ceramide-enriched LDL elicited the downregulation of alkaline ceramidase (*ACER2*)*,* delta desturase sphingolipid 2 (*DEGS2*)*,* ceramide glucosyltransferase enzyme (*UGCG*), serine palmitoyltransferase long chain subunits 1, 2 and 3 (*SPTL1, 2, 3*), and ceramide synthase 6 ((*CERS6*)*,* generates C16 ceramide) (Figure 3A and Table S4). Interestingly, the downregulation of *CERS6* gene expression and the likely decrease in ceramide synthase 6 enzyme suggests a rate limiting process regulating this enzyme.

A key regulatory enzyme in lipid metabolism, phosphotidate phosphatase 2C (*PPAP2C*) was upregulated in response to C16 ceramide-enriched LDL. Additionally, *SMPD1* and *4* which encode lysosomal acid sphingomyelinase and neutral membrane sphingomyelinase, respectively, were also upregulated. Recent findings strongly support that altered circulating sphingolipids are associated with the development of nephropathy in diabetes [15,45] and lupus [41,46]. How different circulating sphingolipids influence renal sphingolipid metabolism and signaling remains to be investigated.

#### 3.2.2. Glycosphingolipids Biosynthesis-Ganglio Series Pathway

Glycosphingolipids are particularly abundant in the kidney and play a critical role in kidney function [4]. It has been established that sphingolipid enzyme replacement therapy ameliorates kidney disease progression in sphingolipid-associated genetic disorders such as Gaucher and Fabry disease [47]. However, whether circulating sphingolipids regulate renal glycosphingolipid metabolism is still unknown. Our data showed that all genes in the glycospingolipid biosynthesis-ganglio series pathway that were induced by C16 ceramide-enriched LDL were upregulated (Figure S3), with ST3 β-galactoside α-2,3-sialyltransferase 1 (*ST3GAL1, 5*) (Figure 3B and Table S5) being the most upregulated gene. ST3GAL1 is a type II membrane protein that catalyzes the transfer of sialic acid from CMP-sialic acid to galactose-containing substrates. This sialyltransferase enzyme is believed to be a target for cancer treatment to prevent metastasis [48]. ST3GAL isoform 5 causes GM3 synthase deficiency, which results in the upregulation of ST3GAL1, 5 in podocytes contributing to kidney diseases associated with changes in renal glycosphingolipid levels [4,5,6].

Additionally, the transcripts of ST6 N-acetylgalactosaminide α-2,6-sialyltransferase 4 (*ST6GALNAC4*) and isoform 6 (*ST6GALNAC6*) were upregulated in response to C16 ceramide-enriched LDL (Figure 3B and Table S5). This enzyme is involved in the synthesis of disialylgalactosylgloboside (*DSGG*) from monosialylgalactosylgloboside (*MSGG*) in the kidney [49], and the effect of its upregulation on renal glycosphingolipid levels and associated kidney diseases may possibly be relevant.

#### 3.2.3. Sphingolipid Signaling Pathway

**Figure 4:**
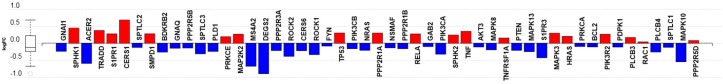
The sphingolipid signaling pathway mRNA transcripts regulated in response to C16 ceramide-enriched LDL in human podocytes. Podocytes were induced with C 16 ceramide-enriched or native LDL and RNA-seq assay was performed as described in Figure 3 legend. Figure generated by iPathwayGuide (Advaita Bioinformatics) software; log FC, logarithm of fold change of gene expression. Box and whisker plot: the ends of the box are the upper and lower quartiles, box spans the interquartile range. Horizontal line inside the box denotes the median and the whiskers are the two lines outside the box extend to the highest and lowest observations.

The sphingolipid signaling pathway genes that were regulated in response to C16 ceramide-enriched LDL are shown in Figure 4, Table S6 and Figure S2. In addition to *SPHK1 & SPHK2*, sphingosine 1 phosphate receptor 1 (*S1PR1*), *CERS1, CERS 6*, mitogen-activated protein kinase 3 (*MAPK3*), ras-related C3 botulinum toxin substrate1 (*RAC1*) and phosphatidylinositol 3-kinase subunit β (*PIK3R2*) were upregulated. In contrast, C16 ceramide-enriched LDL elicited the downregulation of *DEGS2*, *ACER2*, sphingosine 1 phosphate receptor 3 (*S1PR3*), and mitogen-activated protein kinase 8, 10 and 13 (*MAPK 8, 10, 13*). Additionally, phosphatase and tensin homolog (*PTEN*), Rho associated protein kinase2 (*ROCK2*), serine/threonine protein kinase B (*AKT3*) and phosphatidylinositol 4,5 bisphosphate 3-kinase subunit β (*PIK3CB*) were downregulated. It remains to be determined which of these downstream signaling molecules that were induced by circulating sphingolipids could be targeted to modify podocyte pathophysiology.

#### 3.2.4. Adherens Junction Pathway

**Figure 5:**
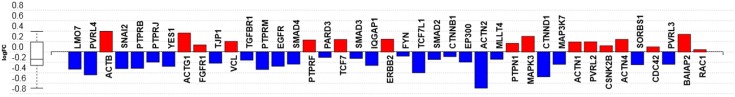
The adherens junction pathway mRNA transcripts regulated in response to C16 ceramide-enriched LDL in human podocytes. Podocytes were induced with C 16 ceramide-enriched or native LDL and RNA-seq assay was performed as described in Figure 3 legend. Figure generated by iPathwayGuide (Advaita Bioinformatics) software; log FC, logarithm of fold change of gene expression. Box and whisker plot: the ends of the box are the upper and lower quartiles, box spans the interquartile range. Horizontal line inside the box denotes the median and the whiskers are the two lines outside the box extend to the highest and lowest observations.

The effects of C16 ceramide-enriched LDL on podocyte gene expression relating to the adherens junction pathway are presented in Figure 5, Table S7, and Figure S4. The glomerular filtration slit (slit diaphragm) is considered as a modified adherens junction [50]. After the metabolic and lysosome pathways, the adherens junction pathway was the third pathway that was significantly affected in response to C16 ceramide-enriched LDL (*p* = 3.3 × 10^−09^) (Table 1). Genes that are involved in encoding the formation of adherens junctions [51] and were downregulated in response to C16 ceramide-enriched LDL include nectin 3 (*PVRL3*), nectin 4 (*PVRL4*), β catenin (*CTNNB1*) and delta catenin (*CTNND1*) (Figure 5 and Table S7). In contrast, nectin 2 (*PVRL2*), and the actin genes *ACTB* and *ACTG1* were upregulated along with the actin crosslinking genes α-actinin 1 and 4 (*ACTN1* and *ACTN4*). It has been shown that mutations in ACTN4 are involved in focal segmental glomerulosclerosis (FSGS), which is a leading cause of kidney failure [52]. Mutations in this protein result in increased affinity for actin binding, the formation of intracellular aggregates, and decreased protein half-life, which—as a result of altering podocyte actin cytoskeleton—may induce toxic effects on podocytes. Notably, the α-actinin (*ACTN2*) gene, which is involved in actin cytoskeleton reorganization, was the most downregulated gene in the adherens junction pathway. The gene encoding the tight junction protein 1/zonula occludens 1 (*TJP1*/*ZO1*) was also downregulated (Figure 5 and Table S7). However, the gene encoding vinculin (*VCL*), another key protein involved in the formation of the tight junction complex was upregulated in response to C16 ceramide-enriched LDL.

Genes encoding the transforming growth factor β 1 (TGF-β1) signaling pathway components such as *TGFbR1* and *SMAD 2, 3, 4* were all downregulated in response to C16 ceramide-enriched LDL (Figure 5 and Table S7). Interestingly, the gene encoding epidermal growth factor receptor (*EGFR*) was downregulated, whereas the gene encoding fibroblast growth factor receptor 1 (*FGFR1*) was upregulated. It has been suggested that such interactions might serve as negative feedback loops to limit the extent of cellular activation [53].

The gene encoding SNAI2—a zinc finger protein commonly known as SLUG—was downregulated in response to C16 ceramide-enriched LDL (Figure 5 and Table S7). This protein is a transcriptional repressor which downregulates the expression of E-cadherin, involved in epithelial-mesenchymal transitions, and has anti-apoptotic activity [54]. The downregulation of this protein might have a positive effect on the continued functionality of healthy podocytes.

#### 3.2.5. MTOR Signaling Pathway

**Figure 6:**
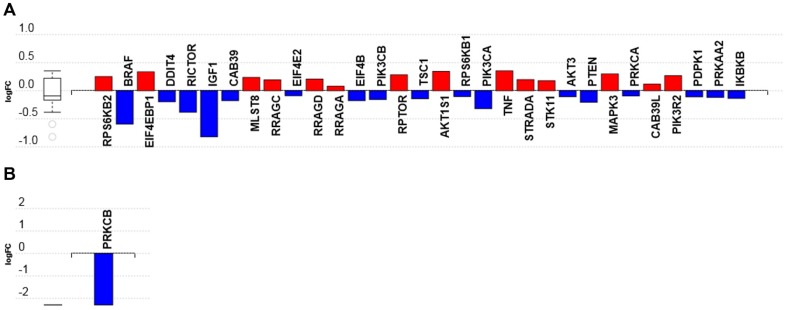
The mTOR signaling pathway mRNA transcripts regulated in response to C16 ceramide-enriched lipoproteins in human podocytes. Podocytes were induced with C 16 ceramide-enriched or native lipoproteins and RNA-seq assay was performed as described in Figure 3 legend. (**A**) Cells induced with ceramide-enriched or native LDL; (**B**) cells induced with ceramide-enriched or native HDL2. Figure generated by iPathwayGuide (Advaita Bioinformatics) software; log FC, logarithm of fold change of gene expression. Box and whisker plot: the ends of the box are the upper and lower quartiles, box spans the interquartile range. Horizontal line inside the box denotes the median and the whiskers are the two lines outside the box extend to the highest and lowest observations.

The treatment of podocytes for 7 h with C16 ceramide-enriched LDL upregulated several intermediates in the mTOR signaling pathway (Figure S5). These include the genes *MAPK3,* the regulatory-associated protein of mTOR (*RPTOR*)*,* proline-rich AKT1 substrate 1 (*AKT1S1*)*,* ribosomal protein S6 kinase β 1 and 2 (*RPS6KB1, 2*) and the proline-rich AKT substrate of 40 kDa (*PRAS40*) (Figure 6A and Table S8). In contrast, several intermediates in the mTOR signaling pathway—including insulin-like growth factor 1 (*IGF1*)*,* phosphoinositide 3 kinase catalytic subunit α (*PIK3CA*, a class I PI3K)*,* the proto-oncogene B-RAF (*BRAF*), *AKT3, PTEN,* tuberous sclerosis 1 (*TSC1*) and the rapamycin-insensitive companion of mammalian target of rapamycin (*RICTOR*)—were downregulated in response to C16 ceramide-enriched LDL (Figure 6A, Table S8, and Figure S5).

When human podocytes were incubated for 7 h with C16 ceramide-enriched HDL2, the only gene that was downregulated (by over two folds) was the protein kinase C β (*PRKCβ*) (Figure 6B, Table S8, and Figure S6). It is possible that after incubation with HDL2 for 7 h, the activation signal went back to background level, whereas that of LDL was still measurable after 7 h. This could be due to the ‘short’ time needed for HDL particle docking to its receptor(s) versus LDL holoparticle endocytosis via the LDL receptor and delivery of its sphingolipid cargo. The mTOR signaling pathway is known to be a major regulatory pathway in the kidney [29,30,31]; therefore, this particular pathway was selected to test the phosphorylated intermediates (Section 3.3 below). Since signaling pathways are activated by phosphorylation and not by gene expression levels per se, the determination of gene regulation was performed at 7 h post incubation with C16 ceramide-enriched lipoproteins, whereas the mTOR signaling pathway multiplex experiment was performed at 2 h post incubation for the early detection of induction effects. How mTOR signaling pathway components induced by circulating sphingolipids affect podocyte pathophysiology certainly warrants further investigation.

#### 3.2.6. Focal Adhesion Pathway

**Figure 7:**
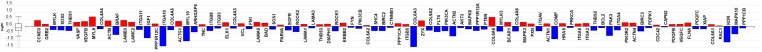
The focal adhesion pathway mRNA transcripts regulated in response to C16 ceramide-enriched LDL in human podocytes. Podocytes were induced with C 16 ceramide-enriched or native LDL and RNA-seq assay was performed as described in Figure 3 legend. Figure generated by iPathwayGuide (Advaita Bioinformatics) software; log FC, logarithm of fold change of gene expression. Box and whisker plot: the ends of the box are the upper and lower quartiles, box spans the interquartile range. Horizontal line inside the box denotes the median and the whiskers are the two lines outside the box extend to the highest and lowest observations.

The focal adhesion-related (cytoskeleton reorganization) genes that were differentially regulated in response to C16 ceramide-enriched LDL treatment of podocytes are shown in Figure 7, Table S9, and Figure S7. The genes encoding filamin (*FLNA*), paxilin (*PXN*), vinculin (*VCL*) and actin (*ACTB*) were all upregulated, which can result in reorganization of focal adhesion and increase cell motility. *Cyclin D*, which regulates cell cycle progression, drives the G1/S phase transition, and is involved in cell growth [55] was also upregulated in response to C16 ceramide-enriched LDL (Figure 7, Table S9). The Rho pathway genes, including Rho-Associated, Coiled-Coil-Containing Protein Kinase 1 and 2 (*ROCK1 and 2*) and Rho GTPase Activating Protein 5 (*ARHGAP5*), which are involved in actin polymerization and the formation of filipodia and lamellipodia were downregulated (Figure 7, Table S9) [56]. In contrast, extracellular matrix (ECM) receptor genes and ECM protein genes including integrin α subunits 4 and V (*ITGA 4, V*), integrin β subunits 3, 8 and A11 (*ITGB 3, 8 and ITGA11*, respectively), non-collagenous ECM protein (*COMP*), collagen 4 α 5 (*COL4A5*), collagen 6 α 3 (*COL6A3*), and collagen 9 α 2 (*COL9A2*) were downregulated in response to C16 ceramide-enriched LDL (Figure 7, Table S9). The downregulation of these ECM proteins suggests that C16 ceramide-enriched LDL treatment may be involved in inducing podocyte dysfunction (e.g., effacement) which needs further investigation. Additionally, the genes encoding the cytokine receptors vascular endothelial growth factor receptor 2 and 3 (*VEGFR 2, 3*), and epidermal growth factor receptor (*EGFR*) were downregulated (Figure 7, Table S9). The effect of these receptors on focal adhesion and migration of endothelial cells has been studied extensively [57]; however, their effects on podocytes in the context of kidney disease processes remains unknown.

#### 3.2.7. Apoptosis Pathway

**Figure 8:**
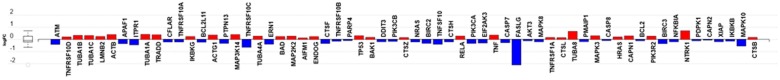
The apoptosis pathway mRNA transcripts regulated in response to C16 ceramide-enriched LDL in human podocytes. Podocytes were induced with C 16 ceramide-enriched or native LDL and RNA-seq assay was performed as described in Figure 3 legend. Figure generated by iPathwayGuide (Advaita Bioinformatics) software; log FC, logarithm of fold change of gene expression. Box and whisker plot: the ends of the box are the upper and lower quartiles, box spans the interquartile range. Horizontal line inside the box denotes the median and the whiskers are the two lines outside the box extend to the highest and lowest observations.

It is well-known that podocyte effacement is a major outcome of glomerular diseases and is associated with podocyte loss leading to renal dysfunction. Whether programmed cell death mechanisms contribute to podocyte loss remains contentious, in part because the detection of apoptosis and other pathways of programmed cell death in podocytes has been technically challenging. Our data show that C16 ceramide-enriched LDL induced the upregulation of the apoptosis-related genes encoding α-tubulin (*TUBA*), *ACTN* and lamin (*LMNA*) (Figure 8, Table S10, and Figure S8), which are known to cause microtubule dysfunction [58], shrinkage of cells [59] and loss of nuclear membrane stability [60], respectively. In contrast, the Fas ligand (*FasLG*) gene, which drives cells into apoptosis [61], was downregulated (Figure 8 and Table S10). Comprehensive understanding of the mechanisms leading to podocyte loss are of particular interest due to the need for developing novel therapeutic strategies targeting these mechanisms to prevent glomerular dysfunction.

### 3.3. C16 Ceramide-Enriched Lipoproteins Regulate Levels of Phosphorylated Intermediates in the MTOR Signaling Pathway

**Figure 9:**
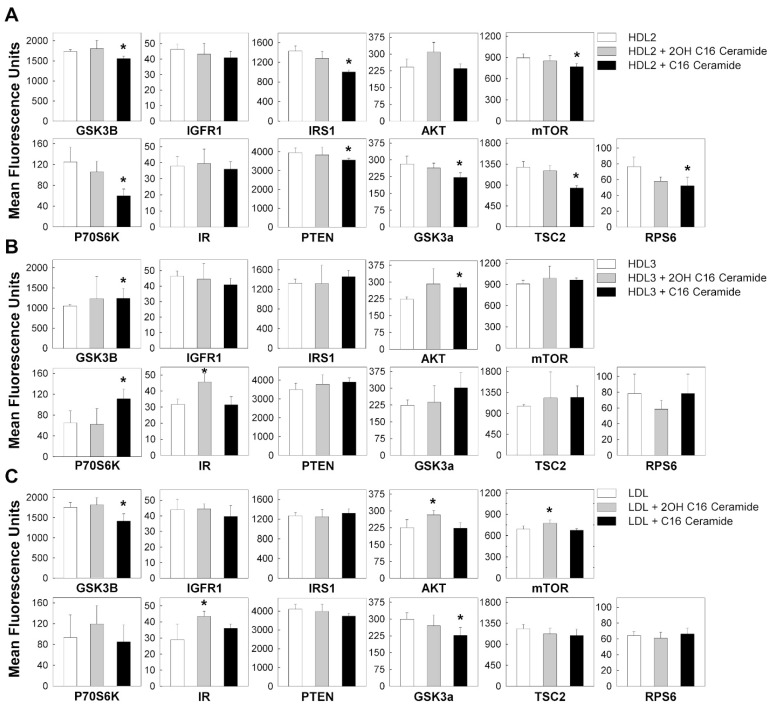
The mTOR signaling pathway phosphorylated proteins regulated in response to C16 ceramide- or 2OH C16 ceramide-enriched lipoproteins in human podocytes. Podocytes were induced with 200 μg/mL ceramide-enriched or with corresponding native lipoproteins (HDL2, HDL3, or LDL), and incubated for 2 h at 37 °C, 5% CO2. Cells were extracted and processed using the mTOR signaling Millipex kit (Millipore). A total of 11.5 µg protein in 25 µL assay buffer was used for each assay sample. The assay was analyzed using the Bio-Plex 200 Multiplex System (Bio-Rad). All treatments were performed in duplicates and the cell extract from each treatment was analyzed in duplicates. (**A**) Treatment with C16 ceramide-enriched HDL2, (**B**) treatment with C16 ceramide-enriched HDL3, (**C**) treatment with C16 ceramide-enriched LDL. Results from treatments with ceramide-enriched lipoproteins were compared to those with corresponding native lipoproteins using Student *t*-test at *p* ≤ 0.05.

The mTOR signaling pathway is a well-described pathway in kidney cell biology, and the phosphorylation of its intermediates is a key step in the activation of the pathway. To further validate our RNAseq data, we employed multiplex technology to detect the regulation (phosphorylation) of mTOR signaling intermediates in response to C16 ceramide-enriched LDL, HDL2 and HDL3 treatments of podocytes. Unlike the qualitative/semi quantitative immunoblot system, the multiplex assay used here offered a quantitative determination of phosphoproteins detected. Each antibody used in this assay was validated and all the antibodies in the provided kits do not cross-react with each other. We found that podocytes treated for two hours with C16 ceramide-enriched HDL2 resulted in downregulation of all phosphorylated intermediates of the mTOR signaling pathway except IGFR1, AKT, and insulin receptor (IR), in comparison to treatment with native HDL2 (Figure 9A). In contrast, C16 ceramide-enriched HDL3 induced the upregulation of GSK3B, AKT and p70S6K phosphorylation, in comparison to treatment with native HDL3 (Figure 9B). It is well-known that not all proteins need to be phosphorylated to be active and that transcript levels do not always translate to protein levels; however, the mTOR pathway after 2 h of incubation with C16 ceramide-enriched HDL2 showed the downregulation of phosphorylation of most activated intermediates measured.

The C16 ceramide-enriched LDL treatment induced downregulation of phosphorylated GSK3a and GSK3B signaling molecules in comparison to treatment with native LDL (Figure 9C). The data thus showed that the effect of C16 ceramide-enriched HDL2 (Figure 9A) and C16 ceramide-enriched LDL (Figure 9C) on the downregulation of phosphorylated GSK3B and GSK3a is similar. This similarity could be attributed to factors relevant to the modification of particle structure and/or size after C16 ceramide-enrichment.

Intriguingly, 2OH C16 ceramide-enriched HDL3 induced upregulation of IR phosphorylation compared to native HDL3, whereas 2OH C16 ceramide-enriched LDL induced upregulation of phosphorylated AKT, mTOR and IR. The pathophysiological relevance of 2OH C16 ceramide modification of lipoproteins is currently unknown.

## 4. Conclusions

The transcriptomics findings of this study suggest that the analyses of sphingolipid profiles of lipoprotein particles, although more laborious, have the potential for an added value over plasma/serum sphingolipidomics in providing more specific information about not only biomarkers of disease but also mechanistic insights about the pathology of the disease and potential drug target molecules. Sphingolipids are non-soluble molecules and the cell membrane is impermeable to these molecules, and in the circulation they are carried and transported by lipoproteins. Mechanistically, our findings suggest that whereas LDL particles—including their bioactive sphingolipid cargo—are likely internalized and metabolized upon their contact with podocytes, HDL particles (mainly HDL2) remain in contact with the cell surface capable of altering the structure of membrane signaling domains and triggering downstream signaling pathways (e.g., mTOR signaling pathway).

For future research on the effect of sphingolipid-altered lipoproteins on the functionality of renal cells (podocytes, mesangial cells, or tubular epithelial cells), other ceramide or sphingolipid species could be incorporated into lipoproteins in vitro, as described here. For sphingolipids containing very long-chain fatty acids (and therefore not easily incorporated in lipoproteins in vitro), fabricated lipoprotein model particles with incorporated sphingolipids could be utilized to investigate the potential for defined sphingolipid species to modify cell membrane sphingolipid composition and alter the cell signaling cascades involved with the pathophysiology of renal cells. Uncovering the role of lipoprotein sphingolipids in the development and progression of kidney disease could completely change the paradigm of intervention to prevent/delay the development or reduce the progression of nephropathy.

## Figures and Tables

**Table 1:** C16 ceramide-enriched LDL regulate gene expression of metabolic and signaling pathways in human podocytes.

Rank	Pathway	*p* Value *	Number of Genes
1	Metabolic pathways	1.1 × 10^−10^	348
2	Lysosome	2.2 × 10^−09^	52
3	Adherens junction	3.3 × 10^−09^	41
4	Ubiquitin mediated proteolysis	5.2 × 10^−08^	50
5	Endocytosis	5.0 × 10^−07^	87
6	Spliceosome	5.6 × 10^−05^	44
7	AGE-RAGE signaling pathway in diabetic complications	0.0003	45
8	Sphingolipid signaling pathway	0.0004	51
12	Insulin signaling pathway	0.0007	57
14	mTOR signaling pathway	0.0008	31
17	Focal adhesion	0.0010	91
18	PI3K-Akt signaling pathway	0.0013	114
20	TNF signaling pathway	0.0018	47

* Bonferroni test-validated *p* values.

## Supplementary Materials

The following are available online at https://www.mdpi.com/2073-4425/11/2/178/s1. Figure S1: The sphingolipid metabolism pathway showing genes regulated in response to C16 ceramide-enriched LDL in human podocytes (iPathwayGuide, Advaita Bioinformatics), Figure S2: The glycosphingolipid biosynthesis-ganglio series pathway showing genes regulated in response to C16 ceramide-enriched LDL in human podocytes (iPathwayGuide, Advaita Bioinformatics), Figure S3: The sphingolipid signaling pathway showing the genes regulated in response to C16 ceramide-enriched LDL in human podocytes (iPathwayGuide, Advaita Bioinformatics), Figure S4: The adherens junction pathway showing the genes regulated in response to C16 ceramide-enriched LDL in human podocytes (iPathwayGuide, Advaita Bioinformatics), Figure S5: The mTOR signaling pathway showing genes regulated in response to C16 ceramide-enriched LDL in human podocytes (iPathwayGuide, Advaita Bioinformatics), Figure S6: The mTOR signaling pathway showing the gene regulated in response to C16 ceramide-enriched HDL2 in human podocytes (iPathwayGuide, Advaita Bioinformatics), Figure S7: The focal adhesion pathway showing genes regulated in response to C16 ceramide-enriched LDL in human podocytes (iPathwayGuide, Advaita Bioinformatics), Figure S8: The apoptosis pathway showing genes regulated in response to C16 ceramide-enriched LDL in human podocytes (iPathwayGuide, Advaita Bioinformatics), Table S1: Metabolic and signaling pathways that were significantly (*p* < 0.05) affected by incubation of human podocytes with C16 ceramide-enriched LDL (iPathwayGuide, Advaita Bioinformatics), Table S2: Metabolic and signaling pathways that were significantly (*p* < 0.05) affected by incubation of human podocytes with C16 ceramide-enriched HDL2 (iPathwayGuide, Advaita Bioinformatics), Table S3: Metabolic and signaling pathways that were significantly (*p* < 0.05) affected by incubation of human podocytes with C16 ceramide-enriched HDL3 (iPathwayGuide, Advaita Bioinformatics), Table S4: The sphingolipid metabolism genes regulated in response to C16 ceramide-enriched LDL in human podocytes (iPathwayGuide, Advaita Bioinformatics), Table S5: The glycosphingolipids synthesis genes regulated in response to C16 ceramide-enriched LDL in human podocytes (iPathwayGuide, Advaita Bioinformatics), Table S6: The sphingolipid signaling pathway genes regulated in response to C16 ceramide-enriched LDL in human podocytes (iPathwayGuide, Advaita Bioinformatics), Table S7: The adherens junction pathway genes regulated in response to C16 ceramide-enriched LDL in human podocytes (iPathwayGuide, Advaita Bioinformatics), Table S8: The mTOR signaling pathway genes regulated in response to C16 ceramide-enriched LDL or HDL2 in human podocytes (iPathwayGuide, Advaita Bioinformatics), Table S9: The focal adhesion pathway genes regulated in response to C16 ceramide-enriched LDL in human podocytes (iPathwayGuide, Advaita Bioinformatics), Table S10: The apoptosis pathway genes regulated in response to C16 ceramide-enriched LDL in human podocytes (iPathwayGuide, Advaita Bioinformatics), Table S11: C16 ceramide LDL responsive genes (iPathwayGuide, Advaita Bioinformatics).
