# Supplementary material for: Transcriptomics Reveal Altered Metabolic and Signaling Pathways in Podocytes Exposed to C16 Ceramide-Enriched Lipoproteins
Source: Genes (Basel). 2020 Feb 7;11(2):178. doi: 10.3390/genes11020178 (PMC7073971; doi:10.3390/genes11020178)
Supplement: Supplementary file 1 [file genes-11-00178-s001.zip › Table S10.docx]

**Table S10.** The apoptosis pathway genes regulated in response to C16 ceramide-enriched LDL in human podocytes

| **Symbol** | **entrez** | **logfc** | **adjpv** |
| --- | --- | --- | --- |
| ATM | 472 | -0.40689 | 0.158868 |
| TNFRSF10D | 8793 | 0.207632 | 0.158868 |
| TUBA1B | 10376 | 0.332772 | 0.158868 |
| TUBA1C | 84790 | 0.314913 | 0.158868 |
| LMNB2 | 84823 | 0.242215 | 0.158868 |
| ACTB | 60 | 0.395952 | 0.16275 |
| APAF1 | 317 | -0.34548 | 0.165875 |
| ITPR1 | 3708 | -0.44236 | 0.165875 |
| TUBA1A | 7846 | 0.410544 | 0.165875 |
| TRADD | 8717 | 0.377502 | 0.165875 |
| CFLAR | 8837 | -0.21122 | 0.165875 |
| TNFRSF10A | 8797 | -0.20915 | 0.173602 |
| IKBKG | 8517 | 0.2376 | 0.177593 |
| BCL2L11 | 10018 | -0.22261 | 0.177593 |
| ACTG1 | 71 | 0.361435 | 0.177996 |
| PTPN13 | 5783 | -0.31847 | 0.188007 |
| MAP3K14 | 9020 | 0.397604 | 0.198731 |
| TNFRSF10C | 8794 | -0.64393 | 0.199081 |
| TUBA4A | 7277 | 0.270201 | 0.208839 |
| ERN1 | 2081 | -0.38761 | 0.211316 |
| BAD | 572 | 0.239356 | 0.215256 |
| MAP2K2 | 5605 | 0.268662 | 0.227272 |
| AIFM1 | 9131 | 0.12386 | 0.229799 |
| ENDOG | 2021 | 0.229646 | 0.234352 |
| CTSF | 8722 | -0.33223 | 0.242961 |
| TNFRSF10B | 8795 | -0.14771 | 0.252241 |
| PARP4 | 143 | -0.0929 | 0.255691 |
| TP53 | 7157 | 0.30307 | 0.256182 |
| BAK1 | 578 | 0.171224 | 0.258472 |
| DDIT3 | 1649 | -0.18755 | 0.259067 |
| PIK3CB | 5291 | -0.1575 | 0.260627 |
| CTSZ | 1522 | 0.152916 | 0.261729 |
| NRAS | 4893 | -0.20432 | 0.261729 |
| BIRC2 | 329 | -0.31678 | 0.266963 |
| TNFSF10 | 8743 | -0.39839 | 0.268795 |
| CTSH | 1512 | -0.19685 | 0.28039 |
| RELA | 5970 | 0.276757 | 0.282368 |
| PIK3CA | 5290 | -0.32073 | 0.28777 |
| EIF2AK3 | 9451 | -0.29516 | 0.290709 |
| TNF | 7124 | 0.352375 | 0.295918 |
| CASP7 | 840 | -0.26165 | 0.29723 |
| FASLG | 356 | -2.09788 | 0.301338 |
| AKT3 | 10000 | -0.10819 | 0.307232 |
| MAPK8 | 5599 | -0.18993 | 0.312346 |
| TNFRSF1A | 7132 | 0.158703 | 0.312829 |
| CTSL | 1514 | 0.146106 | 0.313392 |
| TUBA8 | 51807 | 0.659059 | 0.316815 |
| PMAIP1 | 5366 | -0.18103 | 0.318155 |
| MAPK3 | 5595 | 0.299089 | 0.324634 |

| CASP8 | 841 | -0.09303 | 0.343666 |
| --- | --- | --- | --- |
| HRAS | 3265 | 0.20733 | 0.344562 |
| CAPN1 | 823 | 0.286258 | 0.346896 |
| BCL2 | 596 | -0.10661 | 0.353292 |
| PIK3R2 | 5296 | 0.267102 | 0.353935 |
| BIRC3 | 330 | -0.34354 | 0.355687 |
| NFKBIA | 4792 | -0.19006 | 0.357361 |
| NTRK1 | 4914 | 0.711606 | 0.358337 |
| PDPK1 | 5170 | -0.11028 | 0.359465 |
| CAPN2 | 824 | -0.05166 | 0.361713 |
| XIAP | 331 | -0.18594 | 0.383027 |
| IKBKB | 3551 | -0.13717 | 0.385745 |
| MAPK10 | 5602 | -0.54271 | 0.393127 |
| CTSB | 1508 | 0.165748 | 0.398166 |
