# Supplementary material for: Transcriptomics Reveal Altered Metabolic and Signaling Pathways in Podocytes Exposed to C16 Ceramide-Enriched Lipoproteins
Source: Genes (Basel). 2020 Feb 7;11(2):178. doi: 10.3390/genes11020178 (PMC7073971; doi:10.3390/genes11020178)

**Figure S3.** The sphingolipid signaling pathway showing the genes regulated in response to C16 ceramide-enriched LDL in human podocytes


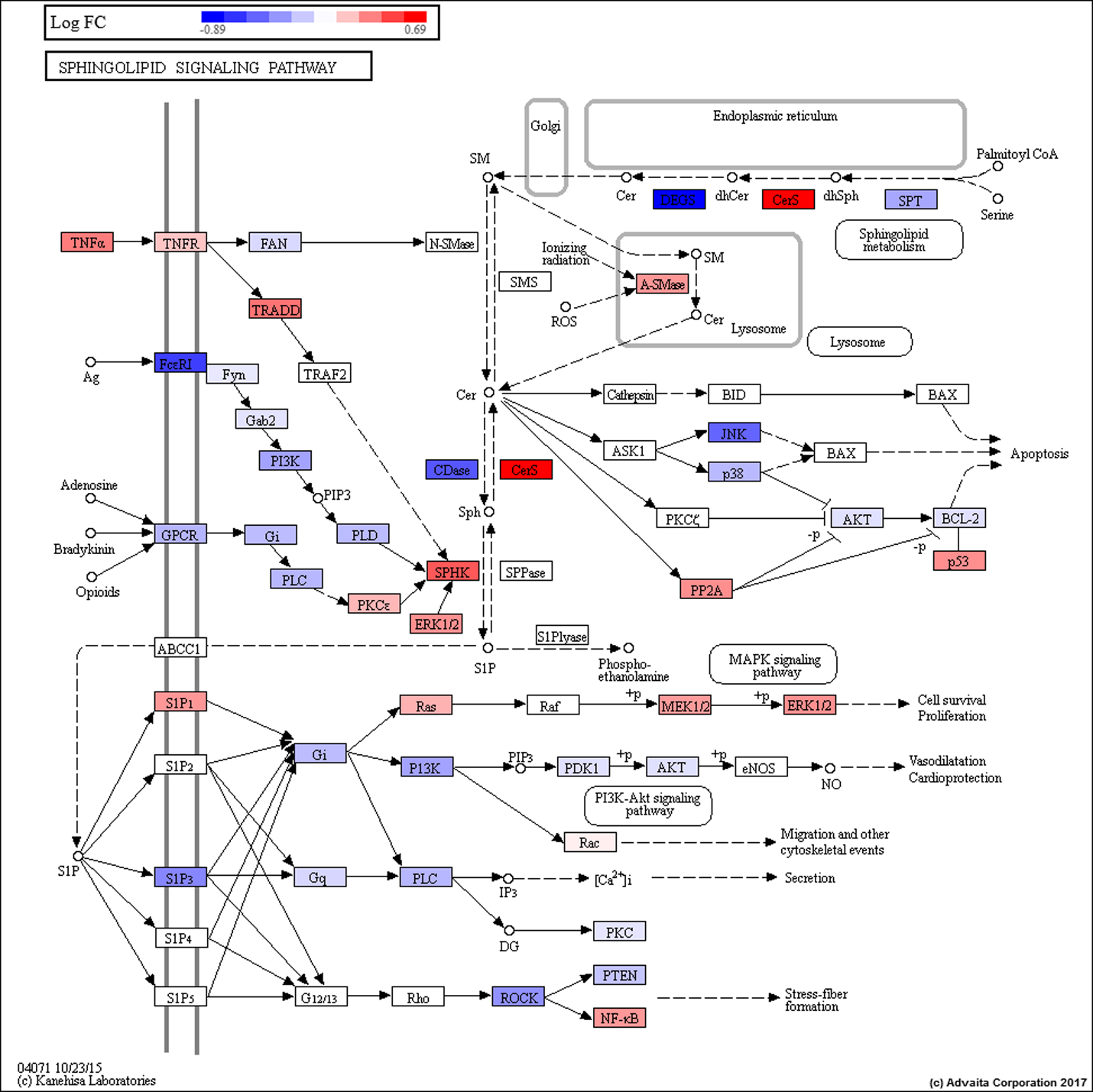

Supplement: Supplementary file 1 [file genes-11-00178-s001.zip › Figure S3.docx]
