# Supplementary material for: Transcriptomics Reveal Altered Metabolic and Signaling Pathways in Podocytes Exposed to C16 Ceramide-Enriched Lipoproteins
Source: Genes (Basel). 2020 Feb 7;11(2):178. doi: 10.3390/genes11020178 (PMC7073971; doi:10.3390/genes11020178)
Supplement: Supplementary file 1 [file genes-11-00178-s001.zip › Table S1.docx]

**Table S1.** Metabolic and signaling pathways that were significantly (p<0.05) affected by incubation of human podocytes with C16 ceramide-enriched LDL

| **pName** | **pv** |
| --- | --- |
| Metabolic pathways | 3.86E-13 |
| Lysosome | 7.62E-12 |
| Adherens junction | 1.14E-11 |
| Ubiquitin mediated proteolysis | 1.77E-10 |
| Endocytosis | 1.70E-09 |
| Spliceosome | 1.91E-07 |
| AGE-RAGE signaling pathway in diabetic complications | 1.26E-06 |
| Sphingolipid signaling pathway | 1.46E-06 |
| Pancreatic cancer | 1.9E-06 |
| Lysine degradation | 1.97E-06 |
| Proteoglycans in cancer | 2.25E-06 |
| Insulin signaling pathway | 2.37E-06 |
| Epstein-Barr virus infection | 2.63E-06 |
| mTOR signaling pathway | 2.8E-06 |
| HTLV-I infection | 3.12E-06 |
| Prostate cancer | 3.43E-06 |
| Focal adhesion | 3.7E-06 |
| PI3K-Akt signaling pathway | 4.61E-06 |
| FoxO signaling pathway | 5.51E-06 |
| TNF signaling pathway | 6.07E-06 |
| Pathways in cancer | 6.23E-06 |
| Pyrimidine metabolism | 6.42E-06 |
| Small cell lung cancer | 7.73E-06 |
| Influenza A | 8.15E-06 |
| Hepatitis C | 9.08E-06 |
| Neurotrophin signaling pathway | 9.31E-06 |
| Pathogenic Escherichia coli infection | 9.38E-06 |
| Huntington's disease | 1.06E-05 |
| Apoptosis | 1.12E-05 |
| Hippo signaling pathway | 1.13E-05 |
