# Supplementary material for: Transcriptomics Reveal Altered Metabolic and Signaling Pathways in Podocytes Exposed to C16 Ceramide-Enriched Lipoproteins
Source: Genes (Basel). 2020 Feb 7;11(2):178. doi: 10.3390/genes11020178 (PMC7073971; doi:10.3390/genes11020178)
Supplement: Supplementary file 1 [file genes-11-00178-s001.zip › Table S2.docx]

**Table S2.** Metabolic and signaling pathways that were significantly (p<0.05) affected by incubation of human podocytes with C16 ceramide-enriched HDL2

| **pName** | **pv** |
| --- | --- |
| Dilated cardiomyopathy | 0.000618567 |
| Drug metabolism - cytochrome P450 | 0.000963645 |
| Regulation of lipolysis in adipocytes | 0.001323073 |
| Steroid hormone biosynthesis | 0.001565545 |
| Tyrosine metabolism | 0.003481503 |
| Metabolism of xenobiotics by cytochrome P450 | 0.005803607 |
| Adrenergic signaling in cardiomyocytes | 0.006997231 |
| Thyroid hormone synthesis | 0.010546999 |
| Renin secretion | 0.010719592 |
| Chemical carcinogenesis | 0.01079904 |
| Retinol metabolism | 0.01238869 |
| Pancreatic secretion | 0.012418143 |
| Phenylalanine metabolism | 0.012874478 |
| Calcium signaling pathway | 0.014925196 |
| Protein digestion and absorption | 0.017372747 |
| Primary immunodeficiency | 0.021161201 |
| Neuroactive ligand-receptor interaction | 0.021331038 |
| Bile secretion | 0.024399241 |
| Renin-angiotensin system | 0.031527859 |
| Histidine metabolism | 0.032905612 |
| Gastric acid secretion | 0.033276476 |
| Porphyrin and chlorophyll metabolism | 0.035038375 |
| Complement and coagulation cascades | 0.041342542 |
| Ascorbate and aldarate metabolism | 0.044572682 |
| Cholinergic synapse | 0.04649083 |
| Drug metabolism - other enzymes | 0.046629728 |
| Arrhythmogenic right ventricular cardiomyopathy (ARVC) | 0.048127164 |
| Vibrio cholerae infection | 0.049607014 |
| Mineral absorption | 0.050695505 |
| mRNA surveillance pathway | 0.053030559 |
