# Supplementary material for: Transcriptomics Reveal Altered Metabolic and Signaling Pathways in Podocytes Exposed to C16 Ceramide-Enriched Lipoproteins
Source: Genes (Basel). 2020 Feb 7;11(2):178. doi: 10.3390/genes11020178 (PMC7073971; doi:10.3390/genes11020178)
Supplement: Supplementary file 1 [file genes-11-00178-s001.zip › Table S3.docx]

**Table S3.** Metabolic and signaling pathways that were significantly (p<0.05) affected by incubation of human podocytes with C16 ceramide-enriched HDL3

| **pName** | **pv** |
| --- | --- |
| Gastric acid secretion | 0.001412 |
| Phagosome | 0.002008 |
| Protein digestion and absorption | 0.002535 |
| Measles | 0.003446 |
| Alanine, aspartate and glutamate metabolism | 0.003544 |
| Hepatitis C | 0.004983 |
| Transcriptional misregulation in cancer | 0.005517 |
| Toxoplasmosis | 0.005699 |
| Complement and coagulation cascades | 0.007668 |
| Cell adhesion molecules (CAMs) | 0.009817 |
| Pathogenic Escherichia coli infection | 0.011961 |
| DNA replication | 0.015417 |
| Glycosphingolipid biosynthesis - globo series | 0.016706 |
| Bile secretion | 0.018679 |
| Pathways in cancer | 0.020119 |
| PI3K-Akt signaling pathway | 0.024563 |
| TNF signaling pathway | 0.024888 |
| Small cell lung cancer | 0.025526 |
| Proteoglycans in cancer | 0.02822 |
| Legionellosis | 0.03044 |
| ECM-receptor interaction | 0.030914 |
| Axon guidance | 0.031557 |
| Influenza A | 0.03184 |
| Fructose and mannose metabolism | 0.03283 |
| Nitrogen metabolism | 0.033246 |
| NF-kappa B signaling pathway | 0.037691 |
| HTLV-I infection | 0.037927 |
| Other glycan degradation | 0.040298 |
| Thyroid hormone synthesis | 0.040628 |
| Glycerophospholipid metabolism | 0.04591 |
