# Supplementary material for: Transcriptomics Reveal Altered Metabolic and Signaling Pathways in Podocytes Exposed to C16 Ceramide-Enriched Lipoproteins
Source: Genes (Basel). 2020 Feb 7;11(2):178. doi: 10.3390/genes11020178 (PMC7073971; doi:10.3390/genes11020178)
Supplement: Supplementary file 1 [file genes-11-00178-s001.zip › Table S4.docx]

**Table S4.** The sphingolipid metabolism genes regulated in response to C16 ceramide-enriched LDL in human podocytes

| **Symbol** | **entrez** | **logfc** | **adjpv** |
| --- | --- | --- | --- |
| SPHK1 | 8877 | 0.449302 | 0.158868 |
| ACER2 | 340485 | -0.59034 | 0.158868 |
| SMPD4 | 55627 | 0.174771 | 0.16435 |
| CERS1 | 10715 | 0.686946 | 0.167128 |
| GBA | 2629 | 0.282663 | 0.173513 |
| SPTLC2 | 9517 | -0.12228 | 0.17405 |
| SMPD1 | 6609 | 0.282263 | 0.175416 |
| SPTLC3 | 55304 | -0.30905 | 0.211572 |
| CERK | 64781 | 0.275192 | 0.215256 |
| PPAP2C | 8612 | 0.322658 | 0.21627 |
| DEGS2 | 123099 | -0.89314 | 0.233283 |
| CERS6 | 253782 | -0.21584 | 0.242961 |
| SPHK2 | 56848 | 0.245266 | 0.295064 |
| UGCG | 7357 | -0.31874 | 0.370284 |
| SPTLC1 | 10558 | -0.11762 | 0.392447 |
