# Supplementary material for: Transcriptomics Reveal Altered Metabolic and Signaling Pathways in Podocytes Exposed to C16 Ceramide-Enriched Lipoproteins
Source: Genes (Basel). 2020 Feb 7;11(2):178. doi: 10.3390/genes11020178 (PMC7073971; doi:10.3390/genes11020178)
Supplement: Supplementary file 1 [file genes-11-00178-s001.zip › Table S5.docx]

**Table S5.** The glycosphingolipids synthesis genes regulated in response to C16 ceramide-enriched LDL in human podocytes

| **Symbol** | **entrez** | **logfc** | **adjpv** |
| --- | --- | --- | --- |
| ST3GAL1 | 6482 | 0.156747 | 0.174041 |
| ST6GALNAC4 | 27090 | 0.310941 | 0.188216 |
| ST3GAL5 | 8869 | 0.323362 | 0.210659 |
| ST6GALNAC6 | 30815 | 0.076807 | 0.310095 |
| B4GALNT1 | 2583 | 0.25083 | 0.353292 |
