# Supplementary material for: Transcriptomics Reveal Altered Metabolic and Signaling Pathways in Podocytes Exposed to C16 Ceramide-Enriched Lipoproteins
Source: Genes (Basel). 2020 Feb 7;11(2):178. doi: 10.3390/genes11020178 (PMC7073971; doi:10.3390/genes11020178)
Supplement: Supplementary file 1 [file genes-11-00178-s001.zip › Table S6.docx]

**Table S6.** The sphingolipid signaling pathway genes regulated in response to C16 ceramide-enriched LDL in human podocytes

| **Symbol** | **entrez** | **logfc** | **adjpv** |
| --- | --- | --- | --- |
| GNAI1 | 2770 | -0.23512 | 0.158868 |
| SPHK1 | 8877 | 0.449302 | 0.158868 |
| ACER2 | 340485 | -0.59034 | 0.158868 |
| TRADD | 8717 | 0.377502 | 0.165875 |
| S1PR1 | 1901 | 0.279132 | 0.167128 |
| CERS1 | 10715 | 0.686946 | 0.167128 |
| SPTLC2 | 9517 | -0.12228 | 0.17405 |
| SMPD1 | 6609 | 0.282263 | 0.175416 |
| BDKRB2 | 624 | -0.25918 | 0.18474 |
| GNAQ | 2776 | -0.14516 | 0.210977 |
| PPP2R5B | 5526 | -0.13729 | 0.211511 |
| SPTLC3 | 55304 | -0.30905 | 0.211572 |
| PLD1 | 5337 | -0.23729 | 0.215216 |
| PRKCE | 5581 | 0.194202 | 0.221965 |
| MAP2K2 | 5605 | 0.268662 | 0.227272 |
| MS4A2 | 2206 | -0.67487 | 0.230816 |
| DEGS2 | 123099 | -0.89314 | 0.233283 |
| PPP2R3A | 5523 | -0.21248 | 0.237296 |
| ROCK2 | 9475 | -0.38442 | 0.237807 |
| CERS6 | 253782 | -0.21584 | 0.242961 |
| ROCK1 | 6093 | -0.33376 | 0.253379 |
| FYN | 2534 | -0.08007 | 0.256182 |
| TP53 | 7157 | 0.30307 | 0.256182 |
| PIK3CB | 5291 | -0.1575 | 0.260627 |
| NRAS | 4893 | -0.20432 | 0.261729 |
| PPP2R1A | 5518 | 0.301362 | 0.264049 |
| NSMAF | 8439 | -0.13229 | 0.264049 |
| PPP2R1B | 5519 | -0.15808 | 0.278069 |
| RELA | 5970 | 0.276757 | 0.282368 |
| GAB2 | 9846 | -0.09672 | 0.284198 |
| PIK3CA | 5290 | -0.32073 | 0.28777 |
| SPHK2 | 56848 | 0.245266 | 0.295064 |
| TNF | 7124 | 0.352375 | 0.295918 |
| AKT3 | 10000 | -0.10819 | 0.307232 |
| MAPK8 | 5599 | -0.18993 | 0.312346 |
| TNFRSF1A | 7132 | 0.158703 | 0.312829 |
| PTEN | 5728 | -0.20671 | 0.315992 |
| MAPK13 | 5603 | -0.24453 | 0.317109 |
| S1PR3 | 1903 | -0.42465 | 0.318155 |
| MAPK3 | 5595 | 0.299089 | 0.324634 |
| HRAS | 3265 | 0.20733 | 0.344562 |
| PRKCA | 5578 | -0.09225 | 0.345353 |
| BCL2 | 596 | -0.10661 | 0.353292 |
| PIK3R2 | 5296 | 0.267102 | 0.353935 |
| PDPK1 | 5170 | -0.11028 | 0.359465 |
| PLCB3 | 5331 | 0.173064 | 0.376464 |
| RAC1 | 5879 | 0.043337 | 0.384833 |
| PLCB4 | 5332 | -0.25361 | 0.389078 |
| SPTLC1 | 10558 | -0.11762 | 0.392447 |
| MAPK10 | 5602 | -0.54271 | 0.393127 |
| PPP2R5D | 5528 | 0.084237 | 0.399242 |
