# Supplementary material for: Transcriptomics Reveal Altered Metabolic and Signaling Pathways in Podocytes Exposed to C16 Ceramide-Enriched Lipoproteins
Source: Genes (Basel). 2020 Feb 7;11(2):178. doi: 10.3390/genes11020178 (PMC7073971; doi:10.3390/genes11020178)
Supplement: Supplementary file 1 [file genes-11-00178-s001.zip › Table S7.docx]

**Table S7.** The adherens junction pathway genes regulated in response to C16 ceramide-enriched LDL in human podocytes

| **Symbol** | **entrez** | **logfc** | **adjpv** |
| --- | --- | --- | --- |
| LMO7 | 4008 | -0.32997 | 0.158868 |
| PVRL4 | 81607 | -0.43968 | 0.158868 |
| ACTB | 60 | 0.395952 | 0.16275 |
| SNAI2 | 6591 | -0.31989 | 0.163751 |
| PTPRB | 5787 | -0.31415 | 0.165875 |
| PTPRJ | 5795 | -0.19504 | 0.169257 |
| YES1 | 7525 | -0.27747 | 0.177253 |
| ACTG1 | 71 | 0.361435 | 0.177996 |
| FGFR1 | 2260 | 0.133078 | 0.18474 |
| TJP1 | 7082 | -0.21651 | 0.18475 |
| VCL | 7414 | 0.200894 | 0.198035 |
| TGFBR1 | 7046 | -0.1582 | 0.208076 |
| PTPRM | 5797 | -0.33661 | 0.211695 |
| EGFR | 1956 | -0.2742 | 0.228497 |
| SMAD4 | 4089 | -0.23257 | 0.229772 |
| PTPRF | 5792 | 0.228608 | 0.245049 |
| PARD3 | 56288 | -0.10451 | 0.248422 |
| TCF7 | 6932 | 0.238472 | 0.252095 |
| SMAD3 | 4088 | -0.12368 | 0.252622 |
| IQGAP1 | 8826 | -0.25889 | 0.253933 |
| ERBB2 | 2064 | 0.242789 | 0.255698 |
| FYN | 2534 | -0.08007 | 0.256182 |
| TCF7L1 | 83439 | -0.39915 | 0.263191 |
| SMAD2 | 4087 | -0.14461 | 0.263531 |
| CTNNB1 | 1499 | -0.0897 | 0.272645 |
| EP300 | 2033 | -0.19349 | 0.301501 |
| ACTN2 | 88 | -0.69368 | 0.305408 |
| MLLT4 | 4301 | -0.14057 | 0.312031 |
| PTPN1 | 5770 | 0.16383 | 0.312031 |
| MAPK3 | 5595 | 0.299089 | 0.324634 |
| CTNND1 | 1500 | -0.47658 | 0.327617 |
| MAP3K7 | 6885 | -0.23759 | 0.332974 |
| ACTN1 | 87 | 0.189747 | 0.338885 |
| PVRL2 | 5819 | 0.191545 | 0.340312 |
| CSNK2B | 1460 | 0.118845 | 0.345739 |
| ACTN4 | 81 | 0.239884 | 0.355155 |
| SORBS1 | 10580 | -0.24391 | 0.359465 |
| CDC42 | 998 | 0.097447 | 0.36077 |
| PVRL3 | 25945 | -0.23712 | 0.370963 |
| BAIAP2 | 10458 | 0.33833 | 0.377202 |
| RAC1 | 5879 | 0.043337 | 0.384833 |
