# Supplementary material for: Transcriptomics Reveal Altered Metabolic and Signaling Pathways in Podocytes Exposed to C16 Ceramide-Enriched Lipoproteins
Source: Genes (Basel). 2020 Feb 7;11(2):178. doi: 10.3390/genes11020178 (PMC7073971; doi:10.3390/genes11020178)
Supplement: Supplementary file 1 [file genes-11-00178-s001.zip › Table S8.docx]

**Table S8.** The mTOR signaling pathway genes regulated in response to C16 ceramide-enriched LDL or HDL2 in human podocytes

| **LDL** | | | |
| --- | --- | --- | --- |
| **Symbol** | **entrez** | **logfc** | **adjpv** |
| RPS6KB2 | 6199 | 0.250819 | 0.158868 |
| BRAF | 673 | -0.59549 | 0.165875 |
| EIF4EBP1 | 1978 | 0.336383 | 0.165875 |
| DDIT4 | 54541 | -0.19746 | 0.165875 |
| RICTOR | 253260 | -0.38416 | 0.165875 |
| IGF1 | 3479 | -0.82188 | 0.169215 |
| CAB39 | 51719 | -0.17631 | 0.196025 |
| MLST8 | 64223 | 0.23648 | 0.206342 |
| RRAGC | 64121 | 0.19351 | 0.215924 |
| EIF4E2 | 9470 | -0.08878 | 0.219555 |
| RRAGD | 58528 | 0.206708 | 0.247284 |
| RRAGA | 10670 | 0.079063 | 0.249461 |
| EIF4B | 1975 | -0.17622 | 0.255951 |
| PIK3CB | 5291 | -0.1575 | 0.260627 |
| RPTOR | 57521 | 0.283597 | 0.26284 |
| TSC1 | 7248 | -0.14376 | 0.262897 |
| AKT1S1 | 84335 | 0.343188 | 0.265727 |
| RPS6KB1 | 6198 | -0.10367 | 0.28039 |
| PIK3CA | 5290 | -0.32073 | 0.28777 |
| TNF | 7124 | 0.352375 | 0.295918 |
| STRADA | 92335 | 0.197647 | 0.300481 |
| STK11 | 6794 | 0.176617 | 0.301338 |
| AKT3 | 10000 | -0.10819 | 0.307232 |
| PTEN | 5728 | -0.20671 | 0.315992 |
| MAPK3 | 5595 | 0.299089 | 0.324634 |
| PRKCA | 5578 | -0.09225 | 0.345353 |
| CAB39L | 81617 | 0.116327 | 0.348191 |
| PIK3R2 | 5296 | 0.267102 | 0.353935 |
| PDPK1 | 5170 | -0.11028 | 0.359465 |
| PRKAA2 | 5563 | -0.12004 | 0.381462 |
| IKBKB | 3551 | -0.13717 | 0.385745 |
| **HDL2** | | | |
| **Symbol** | **entrez** | **logfc** | **adjpv** |
| PRKCB | 5579 | -2.29359 | 0.033969 |
