# Supplementary material for: Transcriptomics Reveal Altered Metabolic and Signaling Pathways in Podocytes Exposed to C16 Ceramide-Enriched Lipoproteins
Source: Genes (Basel). 2020 Feb 7;11(2):178. doi: 10.3390/genes11020178 (PMC7073971; doi:10.3390/genes11020178)
Supplement: Supplementary file 1 [file genes-11-00178-s001.zip › Table S9.docx]

**Table S9.** The focal adhesion pathway genes regulated in response to C16 ceramide-enriched LDL in human podocytes

| **Symbol** | **entrez** | **logfc** | **adjpv** |
| --- | --- | --- | --- |
| CCND3 | 896 | 0.423297 | 0.158868 |
| GRB2 | 2885 | 0.162731 | 0.158868 |
| MYLK | 4638 | -0.24159 | 0.158868 |
| SOS2 | 6655 | -0.2666 | 0.158868 |
| THBS1 | 7057 | -0.3102 | 0.158868 |
| VASP | 7408 | 0.294381 | 0.158868 |
| VEGFB | 7423 | 0.354614 | 0.158868 |
| MYL9 | 10398 | 0.677514 | 0.158868 |
| COL4A4 | 1286 | -0.17917 | 0.162195 |
| ACTB | 60 | 0.395952 | 0.16275 |
| BRAF | 673 | -0.59549 | 0.165875 |
| LAMB3 | 3914 | 0.431177 | 0.165875 |
| LAMC2 | 3918 | 0.300451 | 0.165875 |
| ITGA11 | 22801 | -0.23675 | 0.169172 |
| IGF1 | 3479 | -0.82188 | 0.169215 |
| PPP1R12C | 54776 | 0.179496 | 0.172162 |
| ITGA10 | 8515 | -1.40608 | 0.175957 |
| COL4A5 | 1287 | -0.25511 | 0.175967 |
| ACTG1 | 71 | 0.361435 | 0.177996 |
| MYL10 | 93408 | -1.04504 | 0.17876 |
| ARHGAP5 | 394 | -0.40069 | 0.18474 |
| TNC | 3371 | 0.133342 | 0.184772 |
| ITGB8 | 3696 | -0.31664 | 0.191607 |
| ITGB3 | 3690 | -0.13611 | 0.192658 |
| ELK1 | 2002 | 0.275776 | 0.197724 |
| COL4A3 | 1285 | -0.14347 | 0.197823 |
| VCL | 7414 | 0.200894 | 0.198035 |
| FN1 | 2335 | -0.17819 | 0.199914 |
| LAMA5 | 3911 | 0.295936 | 0.203007 |
| BAD | 572 | 0.239356 | 0.215256 |
| SOS1 | 6654 | -0.20983 | 0.222012 |
| PARVA | 55742 | 0.102351 | 0.225179 |
| EGFR | 1956 | -0.2742 | 0.228497 |
| ROCK2 | 9475 | -0.38442 | 0.237807 |
| LAMA1 | 284217 | 0.152134 | 0.238498 |
| LAMA3 | 3909 | -0.11955 | 0.241036 |
| THBS3 | 7059 | 0.133902 | 0.241036 |
| DIAPH1 | 1729 | 0.166065 | 0.242651 |
| ROCK1 | 6093 | -0.33376 | 0.253379 |
| ERBB2 | 2064 | 0.242789 | 0.255698 |
| FYN | 2534 | -0.08007 | 0.256182 |
| PIK3CB | 5291 | -0.1575 | 0.260627 |
| COL6A2 | 1292 | 0.073388 | 0.262897 |
| SHC4 | 399694 | -0.31617 | 0.265964 |
| BIRC2 | 329 | -0.31678 | 0.266963 |
| CTNNB1 | 1499 | -0.0897 | 0.272645 |
| PPP1CA | 5499 | 0.206434 | 0.272645 |
| ITGB5 | 3693 | 0.148414 | 0.282357 |
| COL6A3 | 1293 | -1.45932 | 0.283029 |
| ZYX | 7791 | 0.402293 | 0.283252 |
| COL9A2 | 1298 | -0.2825 | 0.28402 |

| FLT4 | 2324 | -0.38861 | 0.28777 |
| --- | --- | --- | --- |
| PIK3CA | 5290 | -0.32073 | 0.28777 |
| ACTN2 | 88 | -0.69368 | 0.305408 |
| AKT3 | 10000 | -0.10819 | 0.307232 |
| MAPK8 | 5599 | -0.18993 | 0.312346 |
| PPP1R12A | 4659 | -0.30848 | 0.313995 |
| PTEN | 5728 | -0.20671 | 0.315992 |
| COL6A6 | 131873 | 0.649493 | 0.318155 |
| MYLK3 | 91807 | -0.35139 | 0.31917 |
| BCAR1 | 9564 | 0.287666 | 0.321983 |
| COL4A6 | 1288 | -0.34462 | 0.324276 |
| MAPK3 | 5595 | 0.299089 | 0.324634 |
| PXN | 5829 | 0.181085 | 0.32499 |
| ITGAV | 3685 | -0.33006 | 0.325152 |
| ACTN1 | 87 | 0.189747 | 0.338885 |
| COMP | 1311 | -0.89801 | 0.342603 |
| HRAS | 3265 | 0.20733 | 0.344562 |
| PRKCA | 5578 | -0.09225 | 0.345353 |
| ITGA5 | 3678 | 0.180876 | 0.347509 |
| ITGA3 | 3675 | 0.220829 | 0.347809 |
| THBS2 | 7058 | -0.28588 | 0.348292 |
| BCL2 | 596 | -0.10661 | 0.353292 |
| PAK3 | 5063 | -0.66035 | 0.353383 |
| ITGA4 | 3676 | -0.2657 | 0.353935 |
| PIK3R2 | 5296 | 0.267102 | 0.353935 |
| ACTN4 | 81 | 0.239884 | 0.355155 |
| BIRC3 | 330 | -0.34354 | 0.355687 |
| PDPK1 | 5170 | -0.11028 | 0.359465 |
| CDC42 | 998 | 0.097447 | 0.36077 |
| CAPN2 | 824 | -0.05166 | 0.361713 |
| PDGFB | 5155 | 0.14425 | 0.361876 |
| VEGFC | 7424 | 0.15209 | 0.363262 |
| FLNA | 2316 | 0.255772 | 0.375978 |
| PDGFC | 56034 | -0.17578 | 0.37944 |
| XIAP | 331 | -0.18594 | 0.383027 |
| COL6A1 | 1291 | 0.134728 | 0.383924 |
| RAC1 | 5879 | 0.043337 | 0.384833 |
| KDR | 3791 | -1.12264 | 0.384877 |
| MAPK10 | 5602 | -0.54271 | 0.393127 |
| PPP1CB | 5500 | -0.12613 | 0.397554 |
